# Classical conditioning of faciliatory paired-pulse TMS

**DOI:** 10.1038/s41598-023-32894-w

**Published:** 2023-04-16

**Authors:** Stefan P. Ewers, Timo M. Dreier, Siham Al-Bas, Peter Schwenkreis, Burkhard Pleger

**Affiliations:** Department of Neurology, BG University Clinic Bergmannsheil, Ruhr-University Bochum, Bürkle-de-la-Camp Platz 1, 44789 Bochum, Germany

**Keywords:** Consciousness, Neurological disorders, Neuroscience, Medical research

## Abstract

In this proof-of-concept study, we questioned whether the influence of TMS on cortical excitability can be applied to classical conditioning. More specifically, we investigated whether the faciliatory influence of paired-pulse TMS on the excitability of the human motor cortex can be transferred to a simultaneously presented auditory stimulus through conditioning. During the conditioning phase, 75 healthy young participants received 170 faciliatory paired TMS pulses (1st pulse at 95% resting motor threshold, 2nd at 130%, interstimulus interval 12 ms), always presented simultaneously with one out of two acoustic stimuli. In the test phase, 20 min later, we pseudorandomly applied 100 single TMS pulses (at 130% MT), 50 paired with the conditioned tone—50 paired with a control tone. Using the Wilcoxon-Signed Rank test, we found significantly enhanced median amplitudes of motor evoked potentials (MEPs) paired with the conditioned tone as compared to the control tone, suggesting successful conditioning (*p *= 0.031, responder rate 55%, small effect size of r = − 0.248). The same comparison in only those participants with a paired-pulse amplitude < 2 mV in the conditioning phase, increased the responder rate to 61% (n = 38) and effect size to moderate (r = − 0.389). If we considered only those participants with a median paired-pulse amplitude < 1 mV, responder rate increased further to 79% (n = 14) and effect size to r = − 0.727 (i.e., large effect). These findings suggest increasingly stronger conditioning effects for smaller MEP amplitudes during paired-pulse TMS conditioning. These proof-of-concept findings extend the scope of classical conditioning to faciliatory paired-pulse TMS.

## Introduction

In the present proof-of-concept study, we pursued the question whether the influence of transcranial magnetic stimulation on the human motor cortex can be applied to classical conditioning.

Ivan Petrovich Pavlov (1849–1936) founded classical (“Pavlovian”) conditioning and behavioral learning theory^1^. Before starting his groundbreaking experiments, he observed in dogs, that the owner's footsteps triggered salivation even though there was no food in sight. He suspected that for the dog the sound of footsteps, which was regularly followed by feeding, was associated with eating.

Based on these observations, Pavlov designed a series of experiments in which he repetitively presented dogs the sound of a bell (neutral stimulus) while simultaneously offering food (unconditioned stimulus) to provoke salivation (unconditioned response). After conditioning, the sound of the bell (conditioned stimulus) alone provoked salivation (conditioned response) in the absence of food. Since Pavlov’s groundbreaking findings^1^, many studies showed that various body reactions, such as fear responses^2,3^, or the eyeblink reflex^4–6^ can be classically conditioned.

Eye-blink conditioning is one of the most widely studied form of classical conditioning. It involves the pairing of a conditioned stimulus (usually a tone) to an unconditioned stimulus (air puff). An intact cerebellum, red/interpositus nuclei, as well as motor cortex are required to establish this form of conditioning. Neurons located in the motor cortex fire well in advance of the beginning of eyelid conditioned responses^7^, suggesting that they have a causal relationship with the generation of the conditioned response^8^.

Classical conditioning can even take place if the applied neutral stimulus remains unconscious. Jensen et al., for instance, investigated whether analgesic and hyperalgesic responses can be conditioned by subliminally presented visual stimuli^9^. Successful conditioning of pain modulating responses was indeed independent of the awareness of the conditioned stimulus, emphasizing that consciousness is not required for classical conditioning.

Whether artificially modulated brain activity, evoked by non-invasive brain stimulation, can be classically conditioned remains controversial. Two studies found attenuated TMS-induced motor evoked potentials (MEP) after conditioning single TMS pulses with simultaneously presented visual-acoustic stimuli. In both studies, however, conditioned MEPs could not convincingly be captured when visual-acoustic stimuli were presented alone, without TMS^10,11^.

**Figure 1:**
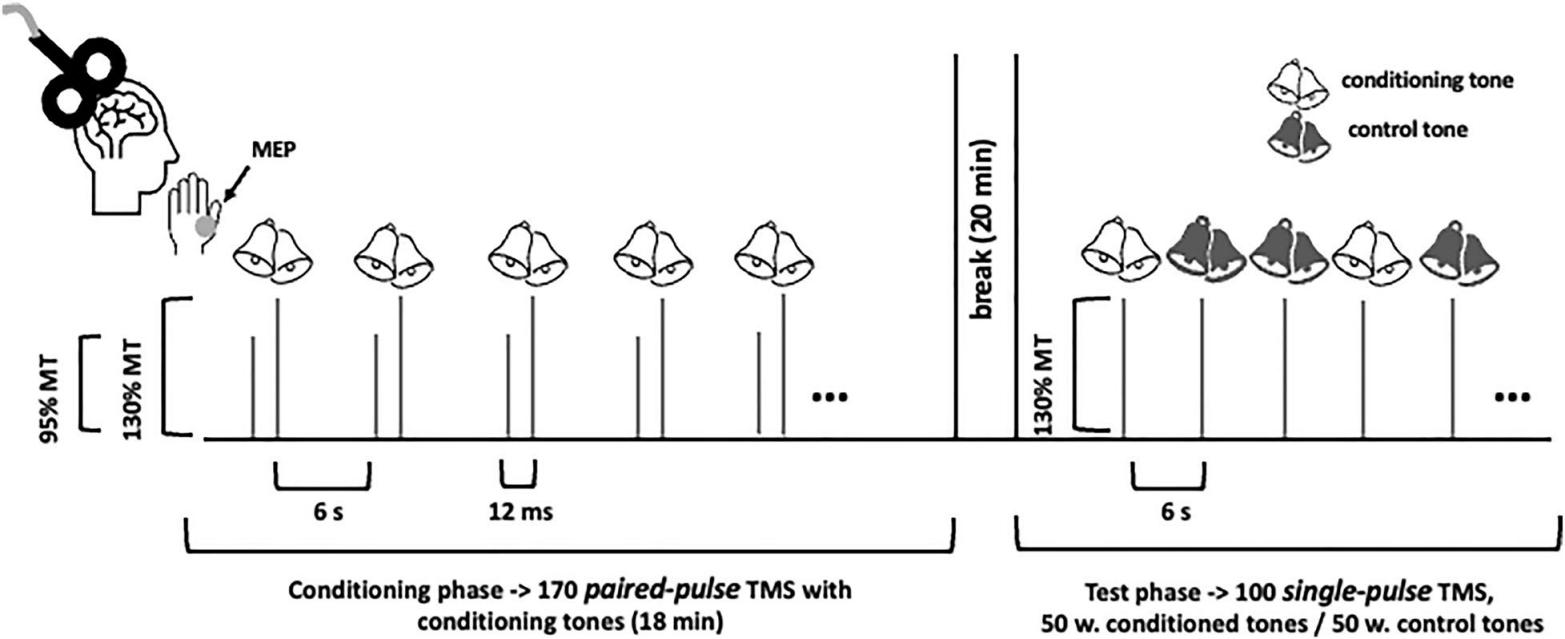
Experimental design. Over the conditioning phase, we applied 170 faciliatory paired TMS pulses to the representation of the right abductor pollices breves muscle over the left primary motor cortex while recording muscle evokes potentials (MEPs) from the target muscle. The first (subthreshold) TMS pulse was adjusted to 95% passive motor threshold (MT), the second (suprathreshold) pulse to 130% passive MT. To induce a faciliatory effect, inter-stimulus interval (ISI) between both TMS pulses was set to 12 ms. Each first subthreshold pulse of each paired-pulse TMS application was paired with one out of two acoustic stimuli (830 Hz or 1480 Hz, counterbalanced across participants). The Intertrial intervals was 6 s long. The conditioning phase was 18 min long. 20 min after the conditioning phase, the test phase started. In the test phase, we applied 100 suprathreshold single TMS pulses with the same 130% MT as used for the second TMS pulse during the conditioning phase. Fifty single TMS pulses were paired with the conditioned tone (white bells) and 50 with the control tone (dark bells). Their order was chosen pseudorandomly. The tone and the single TMS pulse were presented simultaneously as during the conditioning phase. Like in the conditioning phase, inter-trial intervals were set to 6 s. The statistical comparison of single-pulse TMS MEP amplitudes paired with the conditioned tone vs. control tone was used to assess successful conditioning.

In the present proof-of-concept study, we followed another strategy. We used paired-pulse instead of single-pulse TMS and investigated whether its faciliatory influence on the stimulated motor cortex can be applied to classical conditioning. Long intervals between paired TMS pulses (6–20 ms), the first applied at subthreshold intensity and the second at suprathreshold intensity, provoke facilitation of the motor evoked potentials (MEPs) of the target muscle^12–22^. We combined faciliatory paired-pulse TMS, using an interstimulus interval of 12 ms, with a simultaneously presented acoustic stimulus (i.e., simultaneous conditioning). In the following test phase, 20 min later, we then presented single-pulse TMS either together with the conditioned or a control tone (Fig. 1). We expected that single-pulse TMS, paired with the conditioned tone, evokes significantly facilitated MEPs in the target muscle as compared to those MEPs evoked by the same single-pulse TMS but paired with the control tone.

## Results

### Conditioning phase

The resting motor threshold prior to conditioning was assessed at median 43% of maximum stimulator output (1st/3rd quantile 39/47%,). Median MEP amplitudes across participants for the two different tones in the conditioning phase were either 1.83 mV, 1st/3rd quantile 1.31/3.01 mV (830 Hz), or 1.91, 1.15/2.73 mV (1480 Hz) (Mann–Whitney U test, Z = − 0.71, *p *= 0.476, r = − 0.08).

**Figure 2:**
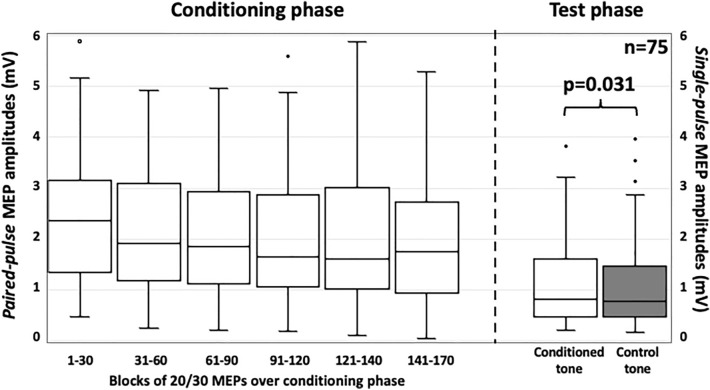
MEP amplitudes (in mV) over the conditioning phase (left boxes) and the test phase (right boxes). Conditioning phase: median MEP amplitudes (grouped in blocks of 20–30 trials) showed no significant shifts over the course of conditioning (Kruskal–Wallis test, chi-square = 0.67, df = 5, *p *= 0.123, eta^2^ = 0.024). Test phase: MEP amplitudes of single TMS pulses paired with the conditioned tone were significantly higher than for the control tone (n = 75, Wilcoxon-Signed Rank, z = − 2.15, *p *= 0.031, r = − 0.248; 55% responder), suggesting successful conditioning of TMS-induced intracortical facilitation. The top of each box indicates the 1st quartile, the bottom the 3rd quartile. The horizontal line within the box marks the median, whiskers indicate minimum and maximum, dots outliers.

The Kruskal–Wallis test revealed no significant shifts of median MEP amplitudes over the course of conditioning phase (chi-square = 0.67, df = 5, *p *= 0.123, eta2 = 0.024; 1st 30 trials: 2.37, 1.35/3.13 mV; 2nd 30 trials: 1.92, 1.18/3.08 mV; 3rd 30 trials: 1.86, 1.13/2.9 mV; 4th 30 trials: 1.65, 1.06/2.86 mV; 5th 20 trials: 1.61, 1.03/2.95 mV; 6th 30 trials: 1.75, 0.94/2.63 mV; Fig. 2).

### Test phase

In the test phase (20 min after conditioning), median MEP amplitudes for the conditioned tone were either 0.69, 0.46/1.15 mV (830 Hz) or 0.91, 0.52/1.96 mV (1480 Hz) (Mann–Whitney U test, z = − 1.02, *p *= 0.307, r = − 0.19). For the control tone median amplitudes were either 0.67, 0.48/1.08 mV (830 Hz) or 0.9, 0.47/2.13 mV (1480 Hz) (Mann–Whitney U test, z = − 1.03, *p *= 0.304, r = − 0.19).

The median MEP amplitude evoked by single TMS pulses paired with the conditioned tone was 0.82, 0.47/1.59 mV (1st 25 trials: 0.84, 0.49/1.64 mV, 2nd 25 trials: 0.79, 0.40/1.72 mV), and 0.77, 0.47/1.43 mV, for the median MEP amplitude paired with the control tone (1st 25 trials: 0.82, 0.52/1.43 mV, 2nd 25 trials: 0.77, 0.36/1.53 mV; n = 75; Fig. 2). The two-sided Wilcoxon-Signed Rank test revealed significantly enhanced amplitudes of MEPs paired with the conditioned tone as compared to those paired with the control tone (z = − 2.15, *p *= 0.031, r = − 0.248; 55% responder (defined by higher MEP amplitude evoked by TMS application with the conditioned as compared to the control tone), 56% in males & 54% in females; Fig. 2), suggesting successful conditioning of TMS-induced facilitation.

Poshoc Wilcoxon-Signed Rank tests were Bonferroni-corrected for multiple comparisons with an adjusted *p*-value of *p *= 0.025. Dividing the 100 single-pulse MEPs into a 1st test phase (1–50 MEPs) and a 2nd phase (51–100 MEPs) revealed no significant differences between conditioned vs. control tone for both, the 1st 50 MEPs (1–50; z = − 1.45, *p *= 0.147, r = − 0.167), and 2nd 50 MEPs (51–100; z = − 1.91, *p *= 0.056, − 0.221). We also found no influences of the two tones on the comparison between conditioned vs. control tone (830 Hz: z = − 1.27, *p *= 0.203, r = − 0.218; 1480 HZ: z = − 1.70, *p *= 0.089, r = − 0.265).

**Figure 3:**
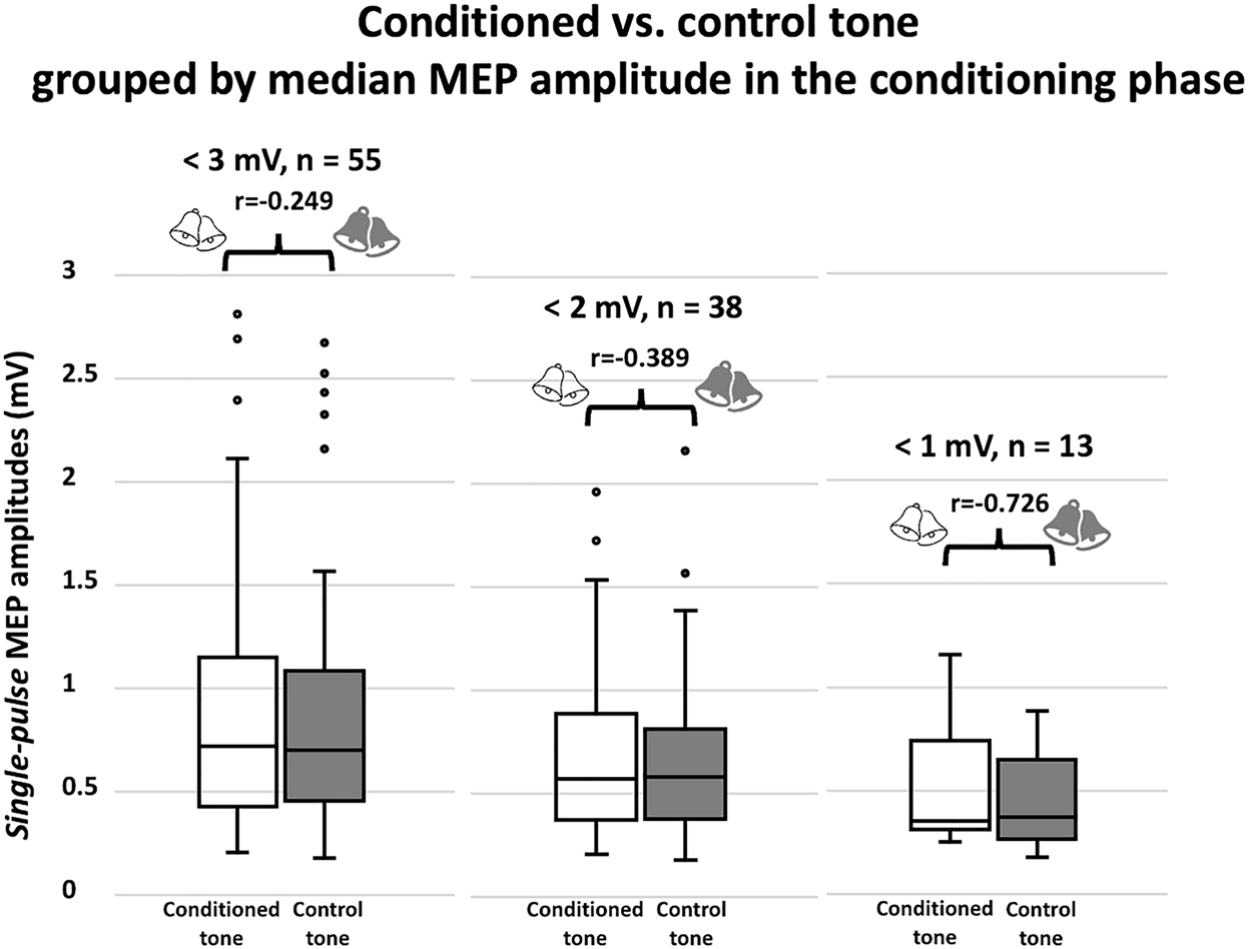
Participant groups according to their median paired-pulse MEP amplitude over the conditioning phase (i.e., < 3 mV, < 2 mV, < 1 mV). For participants with a median paired-pulse MEP amplitude during conditioning < 3 mV (n = 55), the difference between MEP amplitude for conditioned (white box-whiskers) vs. control tone (dark box-whiskers) in the test phase was not significant and the effect size was small (*p *= 0.06, 54% responder rate, r = − 0.249). < 2 mV (n = 38) the responder rate increased to 61% and effect size increased to moderate (r = − 0.389). < 1 mV (n = 13), responder rate increased further to 79% and effect size increased to large (r = − 0.727). These findings suggest that the effect of conditioning related on the height of the paired-pulse MEP amplitudes during the conditioning phase. The top of each box indicates the 1st quartile, the bottom the 3rd quartile. The horizontal line within the box marks the median, whiskers indicate minimum and maximum, dots outliers.

**Figure 4:**
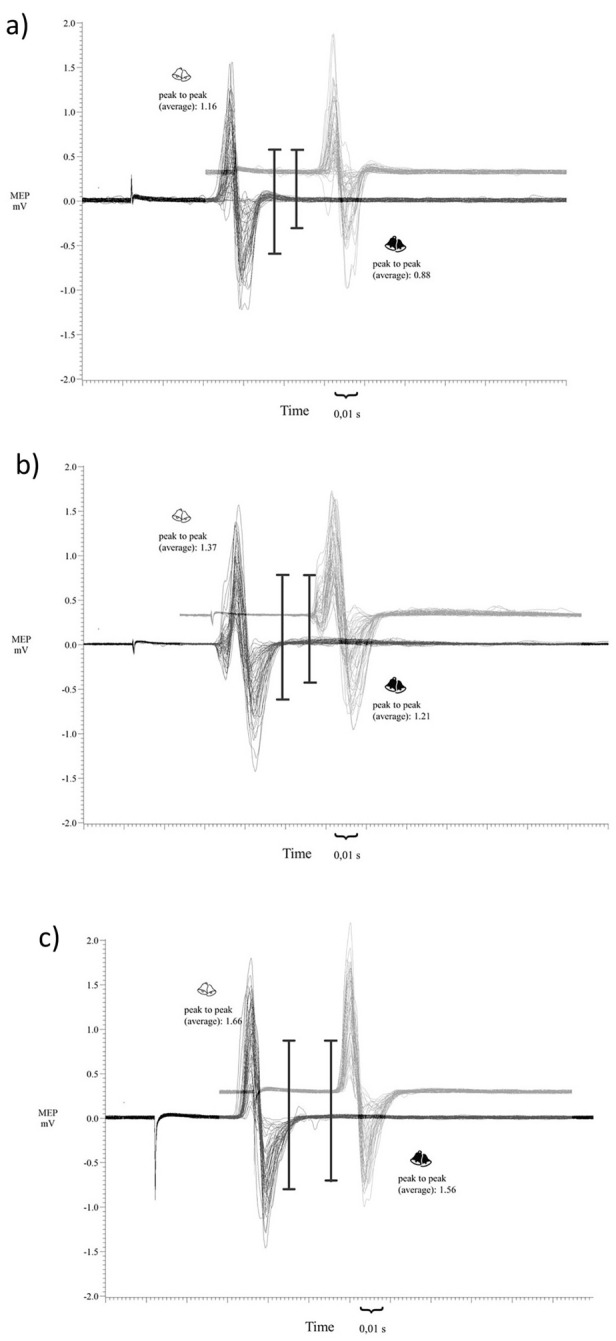
Three exemplary participants—one for each of the three groups (i.e., (**a**) < 1 mV, (**b**) < 2 mV, (**c**) < 3 mV, see Fig. 3). Shown is an overlay of single-pulse MEP responses during the test phase separated into those combined with the conditioned tone (white bells) and those combined with the control tone (dark bells). Vertical lines besides the MEPs index the average across all single-pulse MEP amplitudes during the test phase. Please note that the difference between the MEP combined with the conditioned tone and the MEP with the control tone is strongest for the participant who presented paired-pulse MEP amplitudes during the conditioning phase below 1 mV (**a**). For the participant with paired-pulse MEP amplitudes below 2 mV the conditioning effect became weaker (**b**), whereas the participant with median paired-pulse MEP amplitudes below 3 mV showed the weakest conditioning effect (**c**).

For all participants with a median paired-pulse MEP amplitude during conditioning < 3 mV (n = 55), the difference between the median single-pulse MEP amplitude for conditioned vs. control tone in the test phase was not significant (*p *= 0.06, 54% responder rate; Fig. 3), most likely because of the smaller sample size as compared to the whole sample consisting of 75 participants. Correspondingly, the effect size was small, r = − 0.249. Under 2 mV (n = 38), the proportion of responder increased to 61% (*p *= 0.017) and the effect size increased to moderate (r = − 0.389). Under 1 mV (n = 13), the proportion increased even further to 79% (*p *= 0.005) and the effect size increased to large (r = − 0.727). Exemplary participants for each of the three groups are shown in Fig. 4 ((a) < 1 mV, (b) < 2 mV, (c) < 3 mV).

### Discussion

In the present proof-of-concept study, we raised the question whether the faciliatory influence of paired-pulse TMS on motor cortex excitability can be applied to classical conditioning. To this end, we utilized faciliatory paired-pulse TMS with default parameters^12–22^. The first TMS pulse was applied at subthreshold intensity of 95% resting motor threshold, which conditioned the second pulse, 12 ms later, applied at suprathreshold intensity of 130%. The amount of amplitude amplification after faciliatory paired-pulse TMS is an indirect marker of intracortical facilitation^23^.

Comparing MEPs evoked by single-pulse TMS paired with the conditioned tone to MEPs evoked by the same single-pulse TMS paired with the control tone, we observed significantly enhanced amplitudes, suggesting that TMS-induced facilitation can be classically conditioned to a paired auditory stimulus. The effect size of conditioning across all participants, however, was small (r = − 0.248, 55% responder), but appeared to increase with lower MEP amplitudes during conditioning. In individuals with amplitudes below 2 mV in the conditioning phase, effect size increased to moderate (r = − 0.389) and further to large (r = − 0.727), if the amplitudes were < 1 mV. Together these findings suggest that the effect of conditioning depends on the amplitude of the paired-pulse TMS response during conditioning.

Designed as a proof-of-concept study, we focused on the conditioning effect of paired-pulse TMS, but we did not assess the individual amount of intracortical facilitation and how this related to the individual conditioning effect. In several pilot experiments, we separately tested 50, 100, or 120 paired TMS pulses in different samples consisting of 30 to 50 participants. We only found convincing conditioning effects with the 170 applications we used for the current design. We also tested different lengths of the interval between the conditioning and test phase. Starting the test phase immediately after the conditioning phase, as well as a shorter break of 10 min resulted in increasing fluctuations of MEP amplitudes in the early or later test phase, respectively. A break of 20 min finally stabilized MEP amplitudes. Reliable MEP-recordings over test phase could be achieved with 100 single-pulse TMS applications, not with either 50 or 70. Based on these findings, we had to find the right balance between as many TMS applications as possible—to induce strong conditioning effects and to assess most reliable MEP responses – and as few TMS applications as possible—to avoid an increasing influence of noise (induced by attentional decline and increasing adaptation processes). To achieve this for conditioning phase, we omitted any test measurements of intracortical facilitation prior to or within conditioning. Therefore, it remains unclear whether high paired-pulse MEP amplitudes during conditioning correspond to very weak or very strong facilitatory effects of paired-pulse TMS.

In the former case (i.e., very weak facilitatory effect), MEP responses to interleaved single-pulse TMS would have been in a similar range as the MEP amplitudes due to paired-pulse TMS. In this scenario, transsynaptic activation of most pyramidal cells in the motor cortex by the 1st conditioning TMS stimulus alone might have prevented a relevant facilitatory effect. Such a ceiling effect is well known for intracortical facilitation and has been described by several authors^12–22^. In the context of the present study, single TMS pulses with different stimulation intensities would have allowed us to capture the TMS–response behavior, for example in form of an input–output curve, from weakest to strongest TMS output, with the latter leading to saturated responses indicating the ceiling effect. Alternatively, paired-TMS pulses with increasing intensities of the second test pulse (for example from 95 to 150% resting motor threshold) could have been used to assess the output intensity at which the facilitated MEP response saturates. Future research should include such control measures to describe the association between facilitation and classical conditioning.

A very strong facilitatory effect, as an alternative explanation for weak conditioning effects, could instead rely on an inverse interaction between the brainstem and the motor cortex. The combination of auditory stimuli, activating the startle system, and TMS, applied to the motor cortex, is a well-established paradigm to investigate interactions between the brainstem motor system and the cerebral cortex. An auditory stimulus preceding motor cortex TMS by 30–60 ms was shown to inhibit TMS-induced MEPs in upper limb muscles. The anatomical origin of this interaction seems to depend on intracortical projections within the motor cortex^24^, and inhibitory projections between the caudal brainstem reticular formation and motor cortex^25,26^. If paired-pulse TMS induces very strong facilitation of the pyramidal cells in the motor cortex, it may, at the same time, weaken the influence of auditory inputs in activating cross-modal connections required to link the auditory input to the motor output. Studies, combining TMS conditioning with brain mapping techniques such as EEG or fMRI, are required to deepen our understanding of brainstem-cortex interactions involved in auditory-motor TMS conditioning.

In two previous TMS conditioning studies^10,11^, auditory-visual stimuli were paired with single TMS pulses over the motor cortex to condition TMS evoked MEPs. Without TMS, Luber et al. found that the auditory-visual stimuli alone failed to provoke MEP responses even with repeated conditioning sessions over days^11^. Johnson et al., however, found such MEP responses evoked by auditory-visual stimuli alone, which, however, were much smaller than TMS-induced MEPs and identifiable only in a percentage of tests and participants^10^. TMS preceded by the auditory-visual stimuli, nevertheless, evoked attenuated MEP responses after conditioning in both studies, suggesting that conditioned cortical excitability modifies TMS responsiveness. Our findings generally support these previous findings, however, due to the paired-pulse protocol, we can assign the conditioning effect to a distinct neurophysiological mechanism, namely the facilitation of cortical excitability^12^.

There are many open questions, such as, whether the conditioning effect depends on the individual level of paired-pulse TMS induced intracortical facilitation and how long the conditioned effect lasts before extinction. These questions, as well as the effects of repeated conditioning and pharmacological interventions must be explored in future studies. Whether such conditioning effects mimic the recently described beneficial TMS effects in distinct patient groups^27–30^, remains another challenging question for future research.

Pavlov’s pioneering work inspired decades of conditioning research^1,3–8,31^. More recent findings even suggest that conscious awareness of the neutral stimulus is not required for successful conditioning^9^. Present findings extend the scope of classical conditioning to artificially induced facilitation of cortical excitability through paired-pulse TMS. These findings may motivate further research on conditioning effects of non-invasive brain stimulation of the human brain.

## Methods

### Participants

79 healthy right-handed participants were recruited after signing written informed consent. 3 participants were excluded because of incomplete data. In these participants the session was stopped due to large shifts of the TMS coil during the conditioning phase. Another participant was identified as an outlier with enormous high MEP amplitudes exceeding the sample median plus two times the standard deviation (i.e., median MEP amplitude during conditioning = 8.13 mV, > 5 mV in the following test phase). The data of the remaining 75 participants (48 women, mean age 23 years, 19 to 28) were applied to further analyses. The study was approved by the ethics committee of the medical faculty of the Ruhr University Bochum and conducted in accordance with the Declaration of Helsinki. Exclusion criteria were any neuropsychiatric disorders, any sort of brain damage, metal implants, pregnancy, and permanent medication (contraceptives excluded).

### Experimental setup

Participants were comfortably seated in a chair with armrests and a curved headrest. The backrest was tilted ten to fifteen degrees backwards. After we placed the headphones over participants’ ears (Ear Clip SHS4700/10; Koninklijke Philips N. V., Amsterdam, Netherlands), we fixed the head within the headrest with foam pads that prevented movements to both sides. In pilot experiments, we found that this way of fixation was better than headbands or chin rests. MEPs were recorded from the abductor pollicis brevis (APB) muscle of the right (dominant) hand. MEP electrodes were connected to an amplifier/filter device (D440-2 Isolated Amplifier/Filter; Neurospec AG, Stans, Switzerland). After we identified the MEP hotspot over left M1, we fixed the TMS coils over the hotspot and marked inner and outer edges on the skull with a skin-friendly pen. Paired auditory-TMS stimulations as well as MEP recordings were controlled by a laptop computer (Dell Vostro 3558; Dell Technologies Inc., Round Rock, TX, USA) using the “Micro3” data acquisition unit (Micro1401-3; Cambridge Electronic Design Limited, Cambridge, England) equipped with the “Signal” software (Version 6.05; Cambridge Electronic Design Limited, Cambridge, England).

Two examiners supervised the entire conditioning and test phases. One examiner stood behind the backrest and observed the position of the coil in relation to the markers. The other examiner was sitting at the PC observing each recorded MEP. Whenever MEP amplitude changed from application to application, she/he informed the other examiner to check the coil position. If the participant moved the head, the session was stopped, and the coil repositioned before the session was continued. If the coil could not immediately (within 10 s) be shifted back to the old position, according to the markers on the skull and following MEPs, the session was cancelled. This led to the exclusion of 3 participants (see “Participants”).

After adjusting the coil over participant’s head, we started with the assessment of the resting motor threshold. Afterwards the conditioning phase started. Between conditioning and test phase participants were allowed to stand up, walk around and relax. For test phase, participants were again seated in our examination chair in the same way as for the conditioning phase. We orientated the coil according to the markers on participants’ skull.

### Transcranial magnetic stimulation (TMS)

TMS pulses were applied via a figure-of-eight coil (outside diameter 8.7 cm; peak magnetic field strength 2.2 Tesla; peak electric field strength 660 V/m) connected to two serially linked TMS devices (Magstim BiStim^2^; Magstim Company Ltd, Whitland, South West Wales, UK). We fixed the TMS coil with a tripod stand (Manfrotto 420B; Manfrotto, Cassola, Italy) at the skull over the APB muscle representation on left motor cortex. The TMS coil was orientated at an angle of 45° with respect to the sagittal plane, which induces a posterior–anterior current flow approximately perpendicular to the anterior wall of the central sulcus. The APB representation hot spot was assigned to the point on the skull, where TMS pulses at 50% output power evoked the highest MEP amplitudes. Output power for conditioning was adjusted to the individual resting motor threshold of the APB, defined by the lowest output (in % of the maximum stimulator output) that generated a MEP with a peak-to-peak amplitude of at least 50 µV in at least 5 out of 10 trials in the resting muscle. In the first 22 participants, we additionally tested the resting motor threshold prior to the test phase. Since we found that resting motor thresholds prior to conditioning (median 43% output intensity, 38.5%/46.75% 1st quartile/3rd quartile) and prior to the test phase (44%, 40.25%/46.75%) were almost identical (Wilcoxon-Signed Rank test, z = − 1.17, *p *= 0.243, r = − 0.249), we omitted their reassessment prior to the test phase for the remaining participants.

The interstimulus interval (ISI) between both pulses for paired-pulse TMS was set to 12 ms. The first (subthreshold) pulse was adjusted to 95% resting motor threshold, the second (suprathreshold) pulse to 130% resting motor threshold. Suprathreshold single TMS pulses in the test phase were applied to the same APB representation hot spot on the motor cortex at 130% resting motor threshold.

### Conditioning phase

Conditioning consisted of 170 paired-pulse TMS applications always presented together with one out of two acoustic stimuli, either 830 Hz or 1480 Hz, with a presentation duration of 0.4 s (i.e., simultaneous conditioning, Fig. 1). The assignment of the two acoustic stimuli to either “conditioning” or “control” was counterbalanced across participants. Intertrial intervals were set to 6 s. The session was 18 min long. 20 min after the conditioning phase, the test phase started.

### Test phase

100 single TMS pulses were pseudorandomly applied in the test phase – 50 paired with the conditioned tone and 50 paired with the control tone (either 830 Hz or 1480 Hz) (Fig. 1). The tone and the single TMS pulse were presented simultaneously as during the conditioning phase. Intertrial intervals were also set to 6 s.

### Statistics

First, we questioned whether MEP amplitudes were normally distributed. To this end, we applied the Kolmogorov–Smirnov test. Results were significant for both, the conditioning and test phase (Z = 1.56 and 1.59, *p *= 0.01 and 0.008, respectively) rejecting normal distribution. Based on this result, we applied the median and analyzed data with non-linear tests, including the Wilcoxon-Signed Rank test, the Mann–Whitney U test, and the Kruskal–Wallis test. Significance was set to *p *< 0.05.

We hypothesized that successful conditioning leads to enhanced MEP amplitudes (i.e., conditioned responses) to single TMS pulses during the following test phase, if they are presented with the same tone as used for conditioning. To test this hypothesis, we compared individual median MEP amplitudes in the test phase for the conditioned vs. the control tone with the two-sided Wilcoxon-Signed Rank test in PSPP (version 1.6.2, https://www.gnu.org/software/pspp/get.html). To test whether the effect of conditioning was present only in the 1st 50 (1–50) or the 2nd 50 (51–100) single-pulse TMS applications, we separately applied post-hoc Wilcoxon-Signed Rank test to each of these two blocks. We also tested whether the conditioning effect was different for the two tones using the Mann–Whitney U test. To account for multiple comparisons for these post-hoc tests, we applied Bonferroni correction and adjusted the *p*-value to 0.025.

Stability of paired-pulse MEP amplitudes over the conditioning phase was tested with the Kruskal–Wallis test across 6 trial blocks. Each block consisted of 30/20 trials (i.e., 30/20 paired-pulse TMS applications) (see Fig. 2).

We next questioned whether the effect of conditioning is related to the amplitude of the paired-pulse MEP during conditioning. To this end, we assigned participants according to their median paired-pulse MEP amplitude in the conditioning phase to three groups (i.e., < 3 mV, < 2 mV, < 1 mV). We next compared single-pulse MEP amplitudes for conditioned vs. control tone in the test phase within each of these three groups. Since participants with low amplitudes logically occurred in two or all three groups, we could not apply groups to direct statistical comparisons. The discussion about group differences in based on effect sizes (see next paragraph), remains descriptive and was included to inform future TMS settings (see Discussion for further details).

For Wilcoxon-Signed Rank tests and Mann–Whitney U tests, we calculated the effect size ‘r’, i.e., r = z/$\sqrt{n}$, where ‘z’ is the test statistic, and ‘n’ the sample size. A ‘r’ value of 0.1 indicated a small effect, a value of 0.3 indicated a moderate and a value of 0.5 a large effect. For the Kruskal–Wallis test we calculated the eta squared: eta^2^ = (chi square − k + 1)/(n − k) ; where chi-square is the value obtained in the Kruskal–Wallis test; ‘k’ is the number of groups, and ‘n’ is the total number of observations. The value for eta^2^ ranges from 0 to 1. Values closer to 1 indicate a higher proportion of variance that can be explained by the given variable in the model.

## Data Availability

The data is available on request from the corresponding author (BP).
